# The molecular genetic analysis of the expanding pachyonychia congenita case collection

**DOI:** 10.1111/bjd.12958

**Published:** 2014-08-06

**Authors:** NJ Wilson, EA O'Toole, LM Milstone, CD Hansen, AA Shepherd, E Al-Asadi, ME Schwartz, WHI McLean, E Sprecher, FJD Smith

**Affiliations:** 1 Centre for Dermatology and Genetic Medicine, Colleges of Life Sciences and Medicine, Dentistry & Nursing, University of DundeeDundee, DD1 5EH, U.K; 2 Blizard Institute, Barts and the London School of Medicine and Dentistry, Queen Mary University of LondonLondon, U.K; 3 Department of Dermatology, Yale UniversityNew Haven, CT, U.S.A; 4 Department of Dermatology, University of UtahSalt Lake City, UT, U.S.A; 5 PC ProjectSalt Lake City, UT, U.S.A; 6 Tel Aviv Sourasky Medical CenterTel Aviv, Israel

## Abstract

**Background:**

Pachyonychia congenita (PC) is a rare autosomal dominant keratinizing disorder characterized by severe, painful, palmoplantar keratoderma and nail dystrophy, often accompanied by oral leucokeratosis, cysts and follicular keratosis. It is caused by mutations in one of five keratin genes: *KRT6A*, *KRT6B*, *KRT6C*, *KRT16* or *KRT17*.

**Objectives:**

To identify mutations in 84 new families with a clinical diagnosis of PC, recruited by the International Pachyonychia Congenita Research Registry during the last few years.

**Methods:**

Genomic DNA isolated from saliva or peripheral blood leucocytes was amplified using primers specific for the PC-associated keratin genes and polymerase chain reaction products were directly sequenced.

**Results:**

Mutations were identified in 84 families in the PC-associated keratin genes, comprising 46 distinct keratin mutations. Fourteen were previously unreported mutations, bringing the total number of different keratin mutations associated with PC to 105.

**Conclusions:**

By identifying mutations in *KRT6A*, *KRT6B*, *KRT6C*, *KRT16* or *KRT17*, this study has confirmed, at the molecular level, the clinical diagnosis of PC in these families.

**What's already known about this topic?**

- Pachyonychia congenita (PC) is caused by autosomal dominant mutations in *KRT6A*, *KRT6B*, *KRT6C*, *KRT16* or *KRT17*.
- Plantar pain is the main symptom.
- Palmoplantar keratoderma and nail dystrophy are the predominant characteristics, often accompanied by oral leucokeratosis, cysts and follicular keratosis.

**What does this study add?**

- This study identifies PC-associated keratin mutations in 84 new families with PC recruited by the International Pachyonychia Congenita Research Registry.
- Fourteen of the 46 distinct keratin mutations were previously unreported.
- This study expands the large well-phenotyped and genotyped case series of patients with PC, which is an invaluable resource for the development of mutation-specific and/or gene-specific therapies and for future clinical trials.

Pachyonychia congenita (PC, OMIM #167200 and #167210) is a rare autosomal dominant disorder of keratinization, with hallmark signs of palmoplantar keratoderma (PPK) and nail dystrophy (Fig.1). The main symptom is plantar pain. Additional characteristics include oral leucokeratosis; cysts of various types, including epidermal inclusion cysts and pilosebaceous cysts; follicular keratoses; hoarseness; hyperhidrosis; and natal teeth. The severity and clinical features can vary, as shown in Figure1. While clinical case reports of PC date back to 1906,^1^ and possibly earlier, the understanding behind the genetic basis of PC was not elucidated until 1994,^2^ following discoveries resulting in epidermolysis bullosa simplex (EBS) being the first keratin disorder for which the molecular basis was identified.^3^–^5^ Mutations in one of five keratin genes – *KRT6A*, *KRT6B*, *KRT6C*, *KRT16* or *KRT17* – are now known to cause PC.^6^–^9^ Formerly classified as PC-1 (caused by mutations in *KRT6A* or *KRT16*) and PC-2 (due to mutations in *KRT6B* or *KRT17*), PC nomenclature has recently been revised based on the molecular genetic data; for example, those with mutations in *KRT6A* are named PC-K6a and those with *KRT16* mutations are PC-K16.^10^

**Fig 1:**
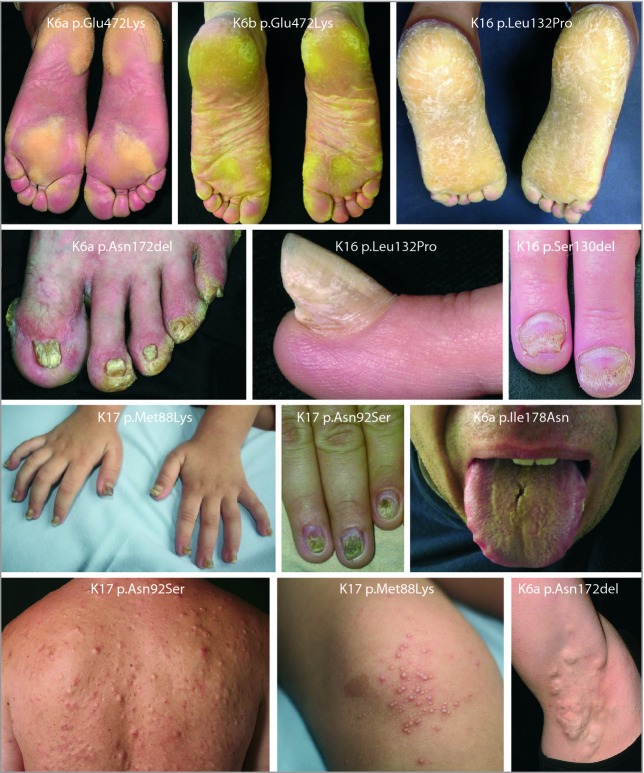
Clinical features of pachyonychia congenita (PC). Palmoplantar keratoderma, nail dystrophy, oral leukokeratosis and cysts in patients with PC with identified mutations.

Keratins comprise the type I and type II groups of the intermediate filament subgroup of cytoskeletal proteins. The 54 known functional keratin genes are divided into epithelial and hair keratins.^11^,^12^ Keratin proteins, albeit very diverse in function, all share a similar protein structure consisting of an α-helical central rod domain comprising four domains (1A, 1B, 2A, 2B) connected by nonhelical regions (L1, L12, L2).^13^ The majority of mutations causing PC are at the start and end of the α-helical rod domain (within 1A and 2B) within the helix boundary motif domains that show evolutionary conservation throughout all intermediate filaments. These motif regions are thought to play a vital role in the interaction of filaments during cytoskeleton construction in forming end–end overlap interactions during filament assembly.^14^

More than 20 keratin genes are now known to cause directly or predispose to fragility syndromes (www.interfil.org), emphasizing the importance of keratins in skin and epithelial appendages. Keratin diseases are almost always inherited in an autosomal dominant manner where mutations act in a dominant negative way, disrupting the ability of keratin filaments to form a strong cytoskeleton, leaving the skin in a fragile, unprotected condition unable to withstand trauma. The mutational spectrum covers all types (missense mutations, small deletion/insertion mutations, splice-site mutations and nonsense mutations), and the disease produced is confined strictly to the epithelial site where the mutant keratin protein is normally expressed; for example, keratin K6a is expressed in palmoplantar epidermis, nail epithelia and mucosal tissues – the tissues affected in PC.

The International Pachyonychia Congenita Research Registry (IPCRR) has, to date, collected clinical and molecular data from more than 500 patients with PC and their families (∼270 families) worldwide. Here, we report molecular analysis of 84 families registered within the last few years of this expanding case collection with a clinical diagnosis of PC. Mutations were identified in *KRT6A*, *KRT6B*, *KRT6C*, *KRT16* or *KRT17*. Forty-six distinct keratin mutations were found, 14 of which were previously unreported mutations, bringing the total number of different keratin mutations associated with PC to 105.

## Materials and methods

Genomic DNA was extracted from saliva collected in an Oragene DNA sample collection kit (DNA Genotek, Kanata, ON, Canada) and extracted according to the manufacturer's protocol or from peripheral blood leucocytes using standard procedures. Samples were obtained by the IPCRR with informed consent and ethical approval from Western Institutional Review Board (IRB), which complies with all principles of the Declaration of Helsinki (Western IRB study no. 20040468).

### Mutation detection

The coding regions of *KRT6A*, *KRT6*, *KRT6C*, *KRT16* and *KRT17* were amplified using primers specific to the respective functional genes and to avoid amplification of K16/K17 pseudogenes. All primers were checked for single nucleotide polymorphisms using Diagnostic SNPCheck (www.ngrl.org.uk/Manchester), and some have been modified since our previous publications to increase specificity (Table S1).^9^,^15^,^16^ For amplification of larger fragments, Takara LA Taq polymerase and buffer (Takara Bio Europe/Clontech, 78100 Saint-Germain-en-Laye, France) was used; for smaller polymerase chain reaction (PCR) reactions, HotStarTaq DNA Polymerase and buffer system (Qiagen, Crawley, U.K.) was used according to the manufacturer's instructions. Specific PCR conditions for each primer set are available on request. PCR products were purified using QiaQuick PCR spin columns (Qiagen) or ExoSAP (using exonuclease 1 and shrimp antartic phosphatase) and sequenced using internal primers on an ABI 3700 Automated DNA sequencing machine (Applied Biosystems, Foster City, CA, U.S.A.). Previously unreported mutations were excluded from at least 90 control DNA samples (180 chromosomes) by sequencing or restriction enzyme digests. The occurrence of sequence changes was also checked on the Exome Variant Server (http://evs.gs.washington.edu/EVS) and dbSNP database.

### RNA extraction

A 3-mm punch biopsy was taken from an affected individual from family 17 with splice-site mutation K6a c.541–2A>G and put in RNA*later* (Life Technologies, Paisley, U.K.). mRNA was extracted using a Quick Prep Micro mRNA purification kit (GE Healthcare UK, Little Chalfont, U.K.) and reverse transcribed using AMV Reverse Transcriptase (Promega, Southampton, U.K.). cDNA was amplified using the Expand High Fidelity PCR system (Roche, Mannheim, Germany) with primers specific for K6a within exon 1 through exon 5.

### Cloning of polymerase chain reaction fragments

The splice-site mutation K6a c.541–2A>G was confirmed by cloning the PCR product derived from cDNA (above) into pCR2.1 vector (TA cloning kit; Life Technologies). Several independent clones were sequenced.

### K16 constructs and transfections

Full-length K16 cDNA was cloned into expression vector pCR3.1 (Life Technologies) and used as a template for site-directed mutagenesis (Stratagene, Cedar Creek, TX, U.S.A.) to make constructs containing K16 p.Arg418Cys and K16 p.Arg418Pro. The three plasmids were transfected individually into PtK2 cells using Lipofectamine 2000 (Life Technologies), according to the manufacturer's protocol. Cells were fixed at 48 and 72 h post-transfection in 1 : 1 methanol : acetone for 5 min, air dried and double-label immunofluorescence was performed. Transfected K16 was detected with rabbit polyclonal antisera (1 : 500 dilution) against human K16 (gift of Pierre Coulombe, Johns Hopkins University, Baltimore, MD, U.S.A.) and the endogenous K8 with neat supernatant monoclonal antibody LE41 to PtK2 K8 (gift of Birgitte Lane, Institute of Medical Biology, Immunos, Singapore). Alexafluor 488 goat anti-mouse and Alexafluor 594 goat anti-rabbit secondary antibodies (Life Technologies) were used at a dilution of 1 : 1000. Nuclei were stained with 4′,6-diamidino-2-phenylindole (DAPI).

## Results

### Clinical details

The main clinical features of individuals involved in this study are reported in Table1. All cases were recruited through the IPCRR, an ongoing research programme to identify patients with PC worldwide and to collect detailed clinical and molecular data from all individuals registered. Autosomal dominant inheritance was observed in 49 of 84 families, while the remaining cases were apparently due to spontaneous mutations. In many cases with previously reported mutations the parents were not screened.

**Table 1:** Mutations and clinical details in new cases of pachyonychia congenita

Family	Mutation – protein change	DNA change	Unreported or known	Familial or spontaneous	Plantar pain	Palmoplantar keratoderma	Thickened nails	Oral leucokeratosis	Cysts and/or follicular hyperkeratosis	Born with teeth?
1	K6a p.Glu163Lys	c.487G>A	Known	Familial	Somewhat painful	PPK	All 20	Yes	NA	No
2	K6a p.Leu170Phe	c.508C>T	Known	Spontaneous	Not painful	PPK, very mild	All 20	No	NA	No
3	K6a p.Asn172del	c.516_518delCAA	Known	Familial	Very painful, but does not use medication	PK	All 20	No	NA	No
4	K6a p.Asn172del	c.516_518delCAA	Known	Familial	Very painful, but does not use medication	PPK	All 20	Yes	NA	No
5	K6a p.Asn172del	c.516_518delCAA	Known	Spontaneous	NA; under 2 years old	NA; under 2 years old	10 fingernails, 2 toenails	Yes	NA	No
6	K6a p.Asn172del	c.516_518delCAA	Known	Spontaneous	Very painful, but does not use medication	PPK	All 20	Yes	Pilosebaceous, follicular hyperkeratosis	No
7	K6a p.Asn172del	c.516_518delCAA	Known	Spontaneous	Very painful, but does not use medication	PPK	All 20	Yes	Steatocystoma, pilosebaceous, follicular hyperkeratosis	No
8	K6a p.Asn172del	c.516_518delCAA	Known	Spontaneous	Somewhat painful	PPK	All 20	Yes	Steatocystoma, follicular hyperkeratosis	No
9	K6a p.Asn172del	c.516_518delCAA	Known	Familial	Not painful	PPK	All 20	Yes	Follicular hyperkeratosis	No
10	K6a p.Asn172del	c.516_518delCAA	Known	Spontaneous	Often requires medication to handle the pain	PPK	All 20	Yes	NA	No
11	K6a p.Asn172del	c.516_518delCAA	Known	Spontaneous	Somewhat painful	PPK	All 20	Yes	NA	No
12	K6a p.Asn172del	c.516_518delCAA	Known	Familial	Often requires medication to handle the pain	PPK	All 20	Yes	Follicular hyperkeratosis	No
13	K6a p.Phe174Ile	c.520T>A	Unreported	Spontaneous	Very painful, but does not use medication	PK	All 20	Yes	Pilosebaceous, follicular hyperkeratosis	No
14	K6a p.Phe174Ser	c.521T>C	Known	Spontaneous	Very painful, but does not use medication	PK	All 20	Yes	NA	No
15	K6a p.Ile178Asn	c.533T>A	Known	Spontaneous	Very painful, but does not use medication	PK	All 20	Yes	Pilosebaceous, follicular hyperkeratosis	No
16	ND	c.541–1G>C	Known	Familial	Often requires medication to handle the pain	PK	6 fingernails, 10 toenails	Yes	NA	No
17	K6a p.Val181_Gln186del	c.541–2A>G	Known	Familial	Somewhat painful	PK	All 20	Yes	Pilosebaceous, follicular hyperkeratosis	No
18	K6a p.Val181_Gln186del	c.541–2A>G	Known	Familial	Very painful, but does not use medication	PPK	All 20	Yes	Pilosebaceous, follicular hyperkeratosis	No
19	K6a p.Val181_Gln186del	c.541–2A>G	Known	Familial	Very painful, but does not use medication	PK	All 20	Yes	NA	No
20	ND	c.541–2A>C	Known	Spontaneous	Somewhat painful	PK	5 fingernails, 10 toenails	Yes	NA	No
21	K6a p.Glu461Ter	c.1381G>T	Unreported	Spontaneous	Very painful, but does not use medication	PK	5 fingernails, 10 toenails	Yes	Steatocystoma, pilosebaceous, follicular hyperkeratosis	No
22	K6a p.Ile462Asn	c.1385T>A	Known	Spontaneous	Very painful, but does not use medication	PK	All 20	Yes	Pilosebaceous, follicular hyperkeratosis	No
23	K6a p.Ala463Pro	c.1387G>C	Known	Spontaneous	Somewhat painful	PPK	All 20	Yes	Follicular hyperkeratosis	No
24	K6a p.Thr464Pro	c.1390A>C	Known	Familial	Very painful, but does not use medication	PK	All 20	Yes	Follicular hyperkeratosis	No
25	K6a p.Leu468Pro	c.1403T>C	Known	Spontaneous	Very painful, but does not use medication	PPK	All 20	Yes	Follicular hyperkeratosis	No
26	K6a p.Leu469Pro	c.1406T>C	Known	Familial	Often requires medication to handle the pain	PPK	All 20	Yes	Steatocystoma, pilosebaceous	No
27	K6a p.Leu469Pro	c.1406T>C	Known	Spontaneous	Often requires medication to handle the pain	PPK	All 20	Yes	Follicular hyperkeratosis	No
28	K6a p.Glu472Lys	c.1414G>A	Known	Spontaneous	Not painful (under 3 years)	PK	All 20	No	Small red bumps and occasional spots on his face, but they could be standard baby acne	No
29	K6a p.Glu472Lys	c.1414G>A	Known	Spontaneous	Very painful, but does not use medication	PPK	All 20	Yes	Steatocystoma, pilosebaceous, follicular hyperkeratosis	No
30	K6a p.Glu473GlyfsTer91	c.1417dupG	Unreported	Spontaneous	Somewhat painful	PPK	10 fingernails, 7 toenails	Yes	NA	No
31	ND	c.1460–1G>C	Unreported	Familial	Often requires medication to handle the pain	PPK	2 fingernails, 10 toenails	No	NA	No
32	K6b p.Asn172del	c.516_518delCAA	Known	Familial	Somewhat painful	PK	10 fingernails, 7 toenails	Yes	NA	No
33	K6b p.Asn172del	c.516_518delCAA	Known	Familial	Very painful, but does not use medication	PPK	6 fingernails, 10 toenails	Yes	Steatocystoma, pilosebaceous	No
34	K6b p.Asn172del	c.516_518delCAA	Known	Familial	Very painful, but does not use medication	PK	6 fingernails, 10 toenails	No	Pilosebaceous, follicular hyperkeratosis	No
35	K6b p.Asn172del	c.516_518delCAA	Known	Familial	Somewhat painful	PPK	8 fingernails, 10 toenails	No	Pilosebaceous, follicular hyperkeratosis	No
36	K6bGlu461Lys	c.1381G>A	Known	Spontaneous	Very painful, but does not use medication	PPK	7 toenails	Yes	Follicular hyperkeratosis	No
37	K6b p.Leu469Arg	c.1406T>G	Unreported	Spontaneous	Somewhat painful	PK	6 toenails	Yes	Follicular hyperkeratosis	No
38	K6b p.Glu472Lys	c.1414G>A	Known	Spontaneous	Very painful, but does not use medication	PK	8 toenails	Yes	NA	No
39	K6b p.Glu472Lys	c.1414G>A	Known	Familial	Somewhat painful	PK	4 toenails	No	Steatocystoma, pilosebaceous, follicular hyperkeratosis	No
40	K6b p.Glu472Lys	c.1414G>A	Known	Spontaneous	Very painful, but does not use medication	PPK	9 toenails	No	Steatocystoma	No
41	K6c p.Glu472Lys	c.1414G>A	Known	Familial	Very painful, but does not use medication	PPK	4 toenails	Yes	Steatocystoma, pilosebaceous	No
42	K6c p.Glu472Lys	c.1414G>A	Known	Familial	Somewhat painful	PK	2 toenails	No	NA	No
43	K16 p.Met121Lys	c.362T>A	Known	Familial	Very painful, but does not use medication	PPK	All 20	No	NA	No
44	K16 p.Met121Thr	c.362T>C	Known	Spontaneous	Very painful, but does not use medication	PK	4 toenails	No	NA	No
45	K16 p.Leu124His	c.371T>A	Known	Familial	Very painful, but does not use medication	PPK	6 fingernails, 10 toenails	No	NA	No
46	K16 p.Leu124His	c.371T>A	Known	Familial	Very painful, but does not use medication	PPK	5 toenails	No	NA	No
47	K16 p.Asn125Asp	c.373A>G	Known	Familial	Very painful, but does not use medication	PPK	10 toenails	No	Pilosebaceous	No
48	K16 p.Asn125Ser	c.374A>G	Known	Spontaneous	Very painful, but does not use medication	PPK	All 20	No	NA	No
49	K16 p.Asn125Ser	c.374A>G	Known	Spontaneous	Somewhat painful; only 6 years old	PK	2 toenails	No	NA	No
50	K16 p.Asn125Ser	c.374A>G	Known	Familial	Very painful, but does not use medication	PPK	3 toenails	Yes	NA	No
51	K16 p.Asn125Ser	c.374A>G	Known	Familial	Somewhat painful	PPK	8 toenails	No	NA	No
52	K16 p.Asn125Ser	c.374A>G	Known	Spontaneous	Somewhat painful	PPK	2 toenails	No	Follicular hyperkeratosis	No
53	K16 p.Asn125Ser	c.374A>G	Known	Familial	Often requires medication to handle the pain	PPK	All 20	Yes	Follicular hyperkeratosis	No
54	K16 p.Arg127Pro	c.380G>C	Known	Familial	Somewhat painful	PPK	All 20	No	Follicular hyperkeratosis	No
55	K16 p.Arg127Gly	c.379C>G	Unreported	Familial	Very painful, but does not use medication	PPK	All 20	No	Steatocystoma	No
56	K16 p.Arg127Cys	c.379C>T	Known	Familial	Often requires medication to handle the pain	PK	5 toenails	No	Follicular hyperkeratosis	No
57	K16 p.Arg127Cys	c.379C>T	Known	Familial	Very painful, but does not use medication	PPK	2 toenails	Yes	NA	No
58	K16 p.Arg127Cys	c.379C>T	Known	Familial	Very painful, but does not use medication	PPK	3 toenails	No	NA	No
59	K16 p.Arg127Cys	c.379C>T	Known	Familial	Very painful, but does not use medication	PPK	None	No	NA	No
60	K16 p.Arg127Cys	c.379C>T	Known	Familial	Very painful, but does not use medication	PK	6 toenails	No	NA	No
61	K16 p.Arg127Ser	c.379C>A	Unreported	Familial	Often requires medication to handle the pain	PK	7 toenails	No	NA	No
62	K16 p.Arg127His	c.381G>A	Unreported	Familial	Very painful, but does not use medication	PPK	4 toenails	Yes	Pilosebaceous, follicular hyperkeratosis	No
63	K16 p.Ser130del	c.389–391delCCT	Known	Familial	Often requires medication to handle the pain	PPK	All 20	No	Follicular hyperkeratosis	No
64	K16 p.Leu132Pro	c.395T>C	Known	Familial	Very painful, but does not use medication	PPK	All 20	No	NA	No
65	K16 p.Arg418Pro	c.1253G>C	Unreported	Familial	Very painful, but does not use medication	PK	2 toenails	No	NA	No
66	K16 p.Arg418_Arg419del	c.1253_1258delGCCGCC	Unreported	Familial	Very painful, but does not use medication	PPK	2 fingernails, 3 toenails	Yes	NA	No
67	K17 p.Met88Lys	c.263T>A	Known	Spontaneous	NA	None (under 3 years of age)	All 20	No	Follicular hyperkeratosis	Yes
68	K17 p.Met88Arg	c.263T>G	Unreported	Spontaneous	Very painful, but does not use medication	PPK	8 fingernails, 10 toenails	Yes	Steatocystoma, pilosebaceous, follicular hyperkeratosis	No
69	K17 p.Asn90_Asp93delinsIle	c.269_278del10insT	Unreported	Familial	Very painful, but does not use medication	PPK	All 20	No	Follicular hyperkeratosis	No
70	K17 p.Leu91Pro	c.272T>C	Unreported	Spontaneous	Very painful, but does not use medication	PPK	All 20	No	Steatocystoma, follicular hyperkeratosis	No
71	K17 p.Asn92Ser	c.275A>G	Known	Familial	Very painful, but does not use medication	PK	All 20	No	Steatocystoma, pilosebaceous, follicular hyperkeratosis	Yes
72	K17 p.Asn92Ser	c.275A>G	Known	Familial	Not painful	PPK	6 fingernails, 10 toenails	No	Steatocystoma, pilosebaceous, follicular hyperkeratosis	Yes
73	K17 p.Asn92Ser	c.275A>G	Known	Spontaneous	Not painful	PPK	6 fingernails, 10 toenails	No	Steatocystoma, pilosebaceous	Yes
74	K17 p.Asn92Ser	c.275A>G	Known	Spontaneous	Often requires medication to handle the pain	PK	10 fingernails, 8 toenails	Yes	Pilosebaceous	No
75	K17 p.Asn92Ser	c.275A>G	Known	Spontaneous	NA	None (under 3 years of age)	All 20	No	NA	Yes
76	K17 p.Asn92Ser	c.275A>G	Known	Familial	Somewhat painful	PPK	6 fingernails, 2 toenails	No	Steatocystoma, pilosebaceous, follicular hyperkeratosis	Yes
77	K17 p.Asn92Ser	c.275A>G	Known	Familial	Often requires medication to handle the pain	PPK	2 fingernails, 10 toenails	No	Pilosebaceous, follicular hyperkeratosis	Yes
78	K17 p.Arg94Ser	c.280C>A	Unreported	Familial	Very painful, but does not use medication	PK	3 toenails	No	Steatocystoma, pilosebaceous, follicular hyperkeratosis	No
79	K17 p.Arg94Cys	c.280C>T	Known	Familial	Very painful, but does not use medication	PPK	4 toenails	No	Steatocystoma, pilosebaceous, follicular hyperkeratosis	No
80	K17 p.Arg94Cys	c.280C>T	Known	Familial	Not painful	NA	1 toenail	No	Steatocystoma	Yes
81	K17 p.Leu95Pro	c.284T>C	Known	Spontaneous	NA; under 2 years old	NA; under 2 years old	All 20	No	NA	Yes
82	K17 p.Leu95Pro	c.284T>C	Known	Familial	Very painful, but does not use medication	PK	All 20	No	Pilosebaceous	Yes
83	K17 p.Leu99Pro	c.296T>C	Known	Familial	Somewhat painful	PPK	All 20	Yes	Follicular hyperkeratosis	No
84	K17 p.Leu99Pro	c.296T>C	Known	Spontaneous	Very painful, but does not use medication	PPK	All 20	No	Steatocystoma, pilosebaceous, follicular hyperkeratosis	Yes

PK, plantar keratoderma; PPK, palmoplantar keratoderma; NA, not applicable; ND, not determined (protein consequences of a splice site mutation not known with certainty).

### Mutation analysis

Of the 84 families, we identified previously unreported heterozygous mutations in 14 of them and known heterozygous mutations in 70 families. The majority were missense mutations, with the remainder being small in-frame deletion, frameshift, nonsense or splice-site mutations in *KRT6A*, *KRT6B*, *KRT6C*, *KRT16* or *KRT17* (Table1).

### Mutations in *KRT6A* (PC-K6a)

Thirty-one families had mutations in *KRT6A* and of the 18 different mutations, four, including a nonsense mutation, were previously unreported. The most commonly reported mutation in PC is K6a p.Asn172del, found in 10 families here, and overall in ∼30% of kindreds with mutations in K6a and in ∼13% of all families with a PC mutation (including published, www.interfil.org, and those reported here).

A novel missense mutation, K6a p.Phe174Ile, was identified in family 13; other amino acid substitutions at this residue have been reported, p.Phe174Ser in at least 12 families, including one in this study, and p.Phe174Cys in one family.^17^

**Fig 2:**
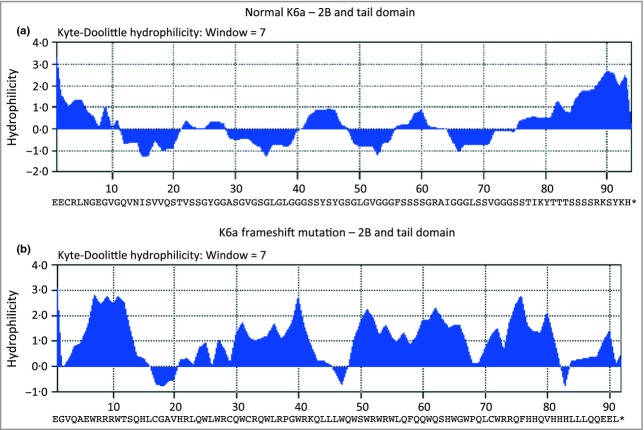
Kyle–Doolittle hydrophilicity analysis of normal and mutant K6a. (a) Normal K6a tail domain consists of alternating hydrophobic and hydrophilic sequences compared with (b) the mutant tail domain, K6a p.Glu473GlyfsTer91, which is mainly hydrophilic.

Mutation K6a p.Glu473GlyfsTer91 (family 30), an unreported frameshift mutation at the end of the 2B domain of K6a, is due to duplication of a single ‘G’ nucleotide, c.1417dupG. This results in a frameshift starting with codon 473, which is changed from glutamic acid to a glycine residue and creates a premature stop codon at position 91 of the new reading frame; this is predicted to cause loss of normal protein function through protein truncation. Specifically, the last 92 correct amino acids are replaced by 90 incorrect amino acids, which are very different in sequence leading to a foreign protein (Fig.2). A previously reported mutation, K6a p.Ser505GlnfsTer59, downstream of the above mutation, also resulted in a 1-bp insertion,^16^ this time in the V2 domain, and the resulting 58 foreign amino acids were the same as in the above mutation.

A previously unreported nonsense mutation, K6a p.Glu461Ter (in Family 21) at the start of the helix termination motif in the 2B domain of K6a, results in a premature stop codon leading to a truncated protein lacking the helix termination motif and tail domain. Missense mutations have previously been reported at this position: K6a p.Glu461, p.Glu461Lys and p.Glu461Gln (www.interfil.org).

Splice-site mutations were found in six families in *KRT6A*. In five of these (families 16–20) mutations were at the intron 1/exon 2 boundary of K6a; three different sequence changes were identified, all of which we have reported previously,^16^ but at that time, owing to the unavailability of mRNA, we were unable to identify the mutations at the protein level. However, in this study we obtained mRNA from a skin biopsy from an affected individual from family 17 with mutation K6a c.541–2A>G. The resulting cDNA was amplified by PCR and cloned, and several clones were sequenced to identify the consequence of this mutation. The mutation is predicted to result in an in-frame deletion of the first six amino acids of exon 2, K6a p. Val181_Gln186del. mRNA was unavailable from other families with the different sequence changes at this splice site. In a sixth family (family 31) a previously unreported splice-site mutation was identified at the exon 8/intron 9 boundary (c.1460–1G>C); unfortunately, we were unable to obtain mRNA from this family to determine the effect of this genomic mutation on RNA splicing.

### Mutations in *KRT6B* (PC-K6b)

Mutations in *KRT6B* were found in nine families, of which one was novel. Only three different mutations have previously been reported in *KRT6B*^17^ (www.interfil.org): p.Asn172del, p.Glu461Lys and p.Glu472Lys. Here, eight of the nine families with mutations in *KRT6B* had mutations at these sites, further indicating that these are mutation hotspot residues in *KRT6B*: p.Asn172del (families 32–35), p.Glu461Lys (family 36) and p.Glu472Lys (families 38–40). Family 37 was found to have an unreported missense mutation within the 2B domain of *KRT6B*, p.Leu469Arg. The analogous mutation has been reported in *KRT6A* (www.interfil.org).

### Mutations in *KRT6C* (PC-K6c)

Only two families (families 41 and 42) had mutations in *KRT6C*, both with the same known mutation, p.Glu472Lys.

### Mutations in *KRT16* (PC-K16)

Mutations in *KRT16* were found in 24 families; the most common recurrent mutations identified were K16 p.Asn125Ser, p.Arg127Cys and p.Leu132Pro (www.interfil.org). Five of the 14 distinct K16 mutations were previously unreported. The 20 or more different mutations reported in keratin 16 include p.Arg127Cys and p.Arg127Pro. In this cohort, we identified previously unreported mutations in three families (families 55, 61 and 62) at K16 p.Arg127: p.Arg127Gly, p.Arg127Ser and p.Arg127His. We also identified one additional family (family 54) with mutation p.Arg127Pro and five families (families 56–60) with p.Arg127Cys. Interestingly, the phenotypic variation seen with these latter two mutations indicates a genotype–phenotype correlation, as proposed in a small study.^18^ Fu *et al*. showed that individuals with mutation p.Arg127Cys were more likely to have milder features of the disorder (milder nail changes, less palmoplantar pain, etc.) than those with amino acid substitution p.Arg127Pro.^18^ Including these additional mutations, ∼24% of reported mutations in *KRT16* occur at this p.Arg127 (www.interfil.org).

Interestingly, a proline substitution for arginine was found to be deleterious in another portion of the helix, whereas a cysteine substitution was not. In Family 65, we identified a new mutation K16 p.Arg418Pro. This mutation in the helix termination motif domain of K16 is predicted to be pathogenic. The unaffected parents of the proband were both wild type. Another sequence change at this position, p.Arg418Cys, which might be predicted to be pathogenic owing to its position within the helix termination motif, is listed on the dbSNP database (minor allele frequency from 1000 Genomes project = 1·9%) and also on the Exome Variant Server (http://evs.gs.washington.edu/EVS/), indicating that it may be a nonpathogenic sequence change. We also found p.Arg418Cys in one of 92 anonymous unrelated control DNA samples and in some members of three unrelated PC families in which known mutations were identified in *KRT6A*, *KRT16* or *KRT17*. To investigate the effect of these sequence changes at K16 p.Arg418, PtK2 cells were transfected with plasmids expressing either wild-type K16, K16 p.Arg418Cys or K16 p.Arg418Pro cDNAs. At 48 h and 72 h post-transfection cells were fixed, and stained with a polyclonal antibody against K16 to detect transfected K16. Endogenous K8 was detected with monoclonal antibody LE41 to PtK2 K8. Nuclei were stained with DAPI. Cells were examined for those containing filaments, filaments plus aggregates or only aggregates. In cells transfected with wild-type K16, 89·5% of transfected cells showed a defined keratin cytoskeleton where wild-type K16 co-localized with endogenous K8; the remaining 10·5% of transfected cells had filaments plus aggregates. Cells transfected with K16 p.Arg418Cys showed a similar pattern to wild-type K16, with 89% of transfected cells with normal filament network and 11% of cells with filaments plus aggregates. However, in cells transfected with K16 p.Arg418Pro, 98·5% of cells contained aggregates and there was collapse/aggregation of the endogenous network; only 1·5% of transfected cells showed a normal filament network (Fig. S1). These results demonstrate that the mutation p.Arg418Pro is disruptive to the normal filament network formation, which, *in vivo*, would result in PC, whereas p.Arg418Cys, *in vitro*, produces a normal filament network similar to wild-type K16 and is therefore unlikely to be pathogenic.

Another novel mutation identified in K16 is a 6-bp in-frame deletion mutation, K16 p.Arg418_Arg419del, in family 66, which results in the deletion of two amino acids from the helix termination motif.

### Mutations in *KRT17* (PC-K17)

*KRT17* mutations were found in 18 families and included four novel mutations. The most common known mutation found was K17 p.Asn92Ser; including previous publications (www.interfil.org) and this study, it occurs in ∼8% of PC families with a confirmed mutation.

Three previously unreported missense mutations were identified in *KRT17*. Family 78 was found to harbour a mutation in K17, p.Arg94Ser. p.Arg94 is a residue already known to be susceptible to mutation owing to several reported cases resulting in amino acid substitutions p.Arg94Cys, p.Arg94His and p.Arg94Pro (www.interfil.org). Two other unreported mutations in K17 were p.Met88Arg (family 68) (mutations have been previously found at this residue p.M88, p.Met88Lys and p.Met88Thr) and p.Leu91Pro (family 70). A novel insertion/deletion mutation in K17, p.Asn90_Asp93delinsIle, found in Family 69, results in deletion of 10 nucleotides and insertion of one nucleotide (T). This leads to an in-frame deletion in a critical region within the helix initiation motif.

## Discussion

The mutations identified in these 84 families add to the increasing data set of the IPCRR of detailed clinical information and corresponding molecular data of individuals with PC. In all cases, the mutations were heterozygous sequence changes – missense, nonsense, small deletion/insertion or splice-site mutations – confirming this is predominantly, if not exclusively, an autosomal dominant disorder. While there are a few case reports of recessive PC reported in the literature,^19^ as yet there are no recessive cases with confirmed genetic testing.

The majority of the mutations identified have been previously reported with some mutations, for example K6a p.Asn172del, K16 p.Asn125Ser, K16 p.Arg127Cys and K17 p.Asn92Ser occurring frequently. For development of mutation-specific forms of treatment, for example small interfering RNA, these residues that are commonly mutated would be obvious targets owing to the larger number of patients that could potentially be treated. More than a third of cases analysed in this study had mutations in K6a, which may partly be reflected by the fact that individuals with K6a mutations tend to present with the most severe features of PC and are therefore more likely to search/ask for support, find the PC Project website (www.pachyonychia.org) and join the IPCRR. Conversely, only two families were identified with mutations in *KRT6C*. In family 42, the proband had for many years been diagnosed with EBS due to blistering of palms and soles and subsequent hyperkeratosis. From the four cases previously reported with *KRT6C* mutations,^17^ together with those in this report, it appears that these individuals present with a milder clinical phenotype than those with mutations in *KRT6A*, *KRT6B*, *KRT16* or *KRT17*. Information regarding the phenotype of those with mutations in *KRT6C* is limited due to the small number of reported cases but this could be explained as only rarely do they come to clinical attention.

**Fig 3:**
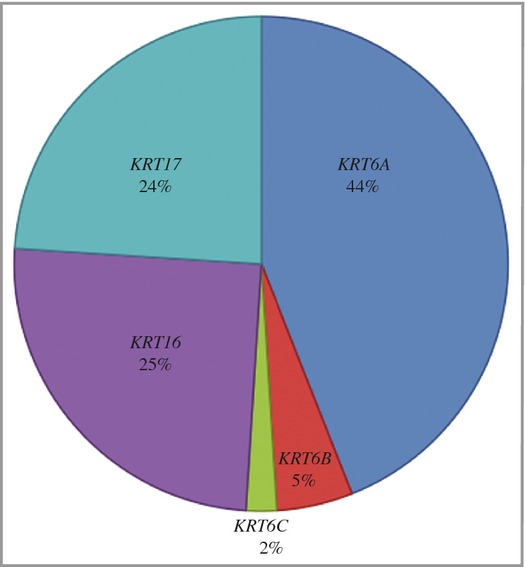
Spectrum of mutations causing pachyonychia congenital (PC), showing the percentage of families in this study and previous publications with mutations in the five keratin genes associated with PC: *KRT6A*, *KRT6B*, *KRT6C*, *KRT16* and *KRT17*.

The results reported here, together with previously published data, are summarized in Figure3, which shows the spectrum of mutations in the five keratin genes associated with PC: *KRT6A*, *KRT6B*, *KRT6C*, *KRT16* and *KRT17*.

The overlapping phenotype of inherited PPKs, independent of their genotype, can make diagnosing them clinically a confusing field.

Individuals presenting with symptoms of PC should undergo genetic analysis for mutations in the five known PC genes (*KRT6A*, *KRT6B* and *KRT6C*, *KRT16* and *KRT17*). In addition, other candidate genes may need to be considered, including *GJB6* encoding connexin 30,^20^ desmoglein 1 (*DSG1*),^21^ desmoplakin (*DSP*),^22^ keratin 9 (*KRT9*),^17^ and frizzled 6,^23^,^24^ mutations in that mimic some features of the PC phenotype and hence should be encompassed into the differential diagnosis of PC. For example, individuals with mutations in connexin, 30 present with nail dystrophy, some PPK and alopecia. The severity of the alopecia varies widely from very subtle to total alopecia but is a key indicator of Clouston syndrome due to mutations in connexin 30, rather than a diagnosis of PC. Autosomal dominant mutations in *DSG1*^21^ and *DSP*, typically result in striate PPK,^25^ although this can also occur in some patients with PC.

Autosomal recessive cases due to desmoplakin mutations also have features that overlap with PC, including palmoplantar blistering and keratoderma with nail dystrophy, which can lead to misdiagnosis. However, these individuals also have distinctive sparse, woolly hair and, importantly, they are at risk of cardiomyopathy. Therefore, diagnosis at the molecular level is important for these individuals in defining the risk of cardiomyopathy and to allow appropriate monitoring of their condition.

Mutations in *FZD6* were recently discovered as the cause of autosomal recessive nail dysplasia.^23^ These individuals present with 20-nail dystrophy from birth or shortly afterwards in the form of thickened, discoloured, claw-shaped nails. There is no involvement of other ectodermal tissues. *FZD6* screening should be considered for any spontaneous cases or known recessive cases with isolated nail dystrophy.

Genetic analysis of the cases in this study has confirmed their clinical diagnosis of PC. A small number of cases within the IPCRR (not reported here) that are clinically not typical of PC have been shown to have mutations in the other genes mentioned above. Correct molecular diagnosis is important to aid in appropriate genetic counselling and patient care. This large, well-phenotyped and genotyped case series is an invaluable resource for the development of mutation-specific and/or gene-specific therapies, and for future clinical trials.

## Supporting Information

Figure S1 Double-label immunofluorescence staining of cells transiently transfected with K16 wild-type and mutant K16. (a) K16 wild-type, (b) K16 p.Arg418Cys and (c) K16 p.Arg418Pro cDNAs in epithelial cell line PtK2. K16 was detected with rabbit polyclonal antisera against human K16 and the endogenous K8 with monoclonal antibody LE41 to PtK2 K8. Nuclei were stained with 4′,6-diamidino-2-phenylindole (DAPI). In cells transfected with wild-type K16, 89·5% of transfected cells showed a defined keratin cytoskeleton where wild-type K16 co-localized with endogenous K8; the remaining 10·5% of transfected cells had filaments plus aggregates. In cells transfected with K16 p.Arg418Cys, similar to wild-type K16, 89% of transfected cells had a normal filament network and 11% of cells had filaments plus aggregates. In contrast, in cells transfected with K16 p.Arg418Pro, 98·5% of cells contained aggregates and there was collapse/aggregation of the endogenous network; only 1·5% of transfected cells showed a normal filament network. Original magnification × 60.

Table S1 Polymerase chain reaction primers for specific amplification of pachyonychia congenita-associated keratin genes.
