# Supplementary figures and images for: The molecular genetic analysis of the expanding pachyonychia congenita case collection
Source: Br J Dermatol. 2014 Aug 6;171(2):343–55. doi: 10.1111/bjd.12958 (PMC4282083; doi:10.1111/bjd.12958)

Supplementary Figure 1

(a) K16 wt

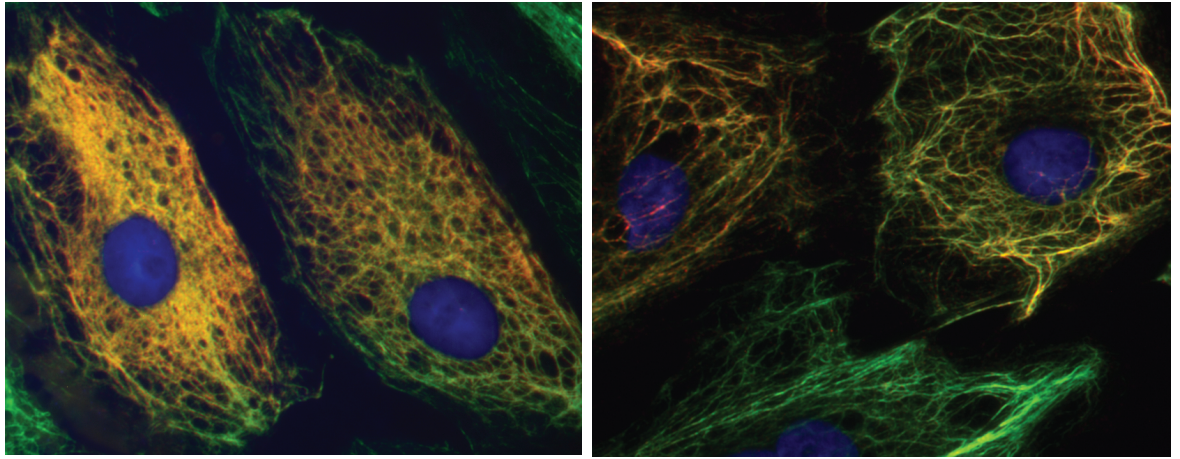

(b) K16 p. Arg418Cys

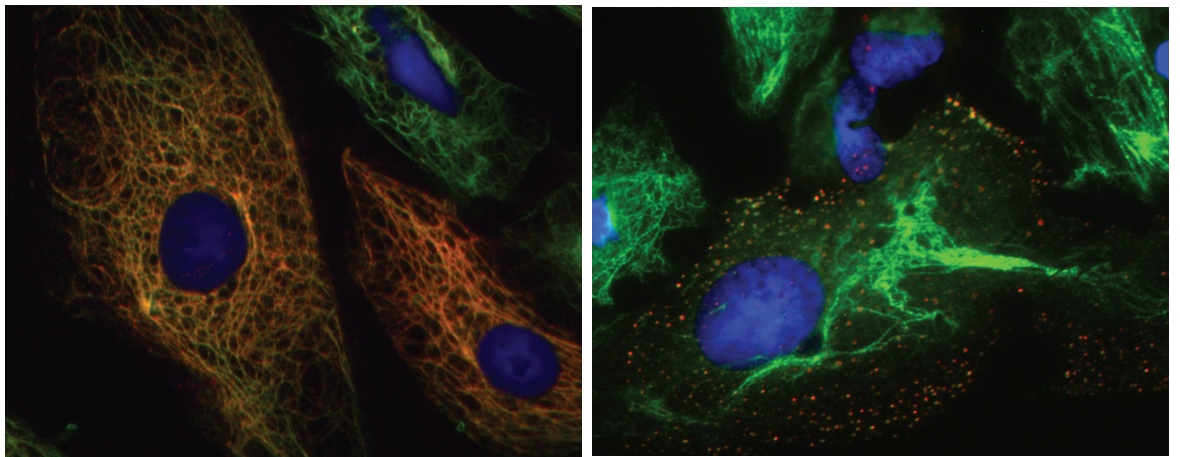

(c) K16 p.Arg418Pro

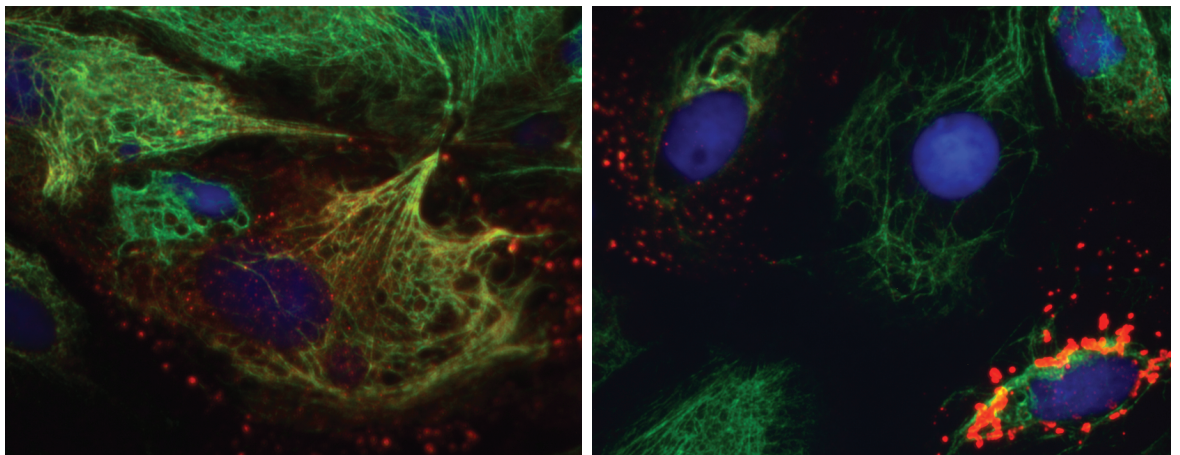

Supplement: Figure S1 — Double-label immunofluorescence staining of cells transiently transfected with K16 wild-type and mutant K16. (a) K16 wild-type, (b) K16 p.Arg418Cys and (c) K16 p.Arg418Pro cDNAs in epithelial cell line PtK2. K16 was detected with rabbit polyclonal antisera against human K16 and the endogenous K8 with monoclonal antibody LE41 to PtK2 K8. Nuclei were stained with 4′,6-diamidino-2-phenylindole (DAPI). In cells transfected with wild-type K16, 89·5% of transfected cells showed a defined keratin cytoskeleton where wild-type K16 co-localized with endogenous K8; the remaining 10·5% of transfected cells had filaments plus aggregates. In cells transfected with K16 p.Arg418Cys, similar to wild-type K16, 89% of transfected cells had a normal filament network and 11% of cells had filaments plus aggregates. In contrast, in cells transfected with K16 p.Arg418Pro, 98·5% of cells contained aggregates and there was collapse/aggregation of the endogenous network; only 1·5% of transfected cells showed a normal filament network. Original magnification × 60. [file bjd0171-0343-SD1.pdf]
