# Supplementary material for: The molecular genetic analysis of the expanding pachyonychia congenita case collection
Source: Br J Dermatol. 2014 Aug 6;171(2):343–55. doi: 10.1111/bjd.12958 (PMC4282083; doi:10.1111/bjd.12958)
Supplement: Table S1 — Polymerase chain reaction primers for specific amplification of pachyonychia congenita-associated keratin genes. [file bjd0171-0343-SD3.docx]

Supplementary Table 1

PCR primers for specific amplification of PC associated keratin genes

| Gene | Primers | Size |
| --- | --- | --- |
| K6a P1914-P6R  Exon 1-6 | P1914 5’ TACCCGGGCTTATTGTGTTAGGAT 3’  P6R 5’ TGTTTGTGTCATGCCATAGGTAGG 3’ | 3729 bp |
| K6a sp2L-sp2R2  Exon 7-9 | SP2L 5’ ACTCCAAACAAACCAGCAGG 3’  SP2R2 5’ CAGATGCCTCCCACTCCATT 3’ | 1896 bp |
| K6b P1888-P5347  Exon 1-6 | P1888 5’ AGCCCAGCCCTTCCCAACC 3’  P5347 5’ ATAAGCCCTTCCAATGCCTCTA 3’ | 3660 bp |
| K6b P5586-P7567  Exon 7-9 | P5586 5’ AGTTGGGCAAGATGTCAGGTCAGA 3’  P7567 5’ AGCAGCAGTTCCCCAAAGATAAGA 3’ | 1982 bp |
| K6c P2467-P6626  Exon 1-6 | P2467 5’ TTGTCCTGCTTCTCCTGCCTCTCG 3’  P6626 5’ GGAAGTCGCGTCAGTTACCTACCT 3’ | 4160 bp |
| K6c P6098-P7641  Exon 7-9 | P6098 5’ CAAACCGAGCATTCATCTTCCATA 3’  7641 5’ GGGGCGGGGGTTCACAATACT 3’ | 1544 bp |
| K16 P1-P2  Exon 1-4 | P1 5’ CACAGCACGCTCTCAGCCTT 3’  P2 5’ CTGCGGTTTTTCTCTGCCAT 3’ | 1672 bp |
| K16 P3-P4  Exon 4-8 | P3 5’ GAGGCTGGTTTGGTGGGGTT3’  P4 5’ CAGCAGAGGAGCAGGGGAGA3’ | 1500 bp |
| K17 F5-P5145  Exon 1-5 | F5 5’ AACCCCGGCAGCCCTACACAACTT 3’  P5145 5’ GGATGGTCAATGCCCTCTTCTG 3’ | 3678 bp |
| K17 6F-P6643  Exon 6-8 | 6F 5’ AGGGATCCAGGCTCACACC 3’  P6643 5’ CAGGGGCCAGAACAAGGACACATT 3’ | 1721 bp |
